# Socioeconomic inequalities in overweight and obesity among South Asian women, 2000–2022

**DOI:** 10.1080/16549716.2026.2736978

**Published:** 2026-09-28

**Authors:** Miranda Hendry, Michael Dibley, Tanvir Huda

**Affiliations:** Faculty of Medicine and Health, University of Sydney School of Public Health, Camperdown, Australia

**Keywords:** Sustainable Development Goal 10: reduced inequalities, women of reproductive age, South Asia, non-communicable disease, nutrition transition, overight, obesity

## Abstract

**Background:**

South Asia is faced with the growing burden of malnutrition and non-communicable diseases, with increasing rates of overweight and obesity. Yet there is limited comparable evidence from repeated Demographic Health Surveys anthropometric data across multiple South Asian countries on the extent to which these trends are associated with social determinants.

**Objectives:**

This study examines the absolute and temporal changes in the prevalence of overweight and obesity among South Asian women, investigating their associations with social determinants in selected South Asian countries that have comparable Demographic Health Survey data.

**Methods:**

We analysed the absolute changes from 2000 until 2022 using log regression model fitted with social determinants: wealth, education, and urban-rural residence. The data was based on Demographic Health Surveys’s from 1,561,090 women aged 15–49 across Bangladesh, India, Nepal, Maldives, and Pakistan.

**Results:**

Overweight and obesity rose significantly in all countries, except the Maldives, with prevalence ranging from 5% to 37%. It was more pronounced among urban (13% to 46% in Bangladesh), less educated (7.3% in India to 70% in the Maldives), and wealthier women (23% in Nepal to 73% in Pakistan).

**Conclusion:**

Overall, the findings indicate a marked epidemiological shift towards increasing prevalence of overweight and obesity rates among South Asian women of reproductive age.

## Paper Context

- **Main findings:** In this paper, we show that the prevalence of overweight and obesity among women of reproductive age in South Asia has generally increased significantly over the past two decades from 2000 up until 2022.
- **Added knowledge:** Using Demographic Health Survey data, we investigated their associations with social determinants including place of residence, education level and wealth quintile and discovered that the increase in body mass index levels are more pronounced among women living in rural areas, with wealthier and holding secondary or higher education level with the fastest rate of increase found as 15% in Bangladesh among poorest communities.
- **Global health impact for policy and action:** This paper presents strong evidence in nutritional epidemiology from robust secondary data to present the impact on increasing weight status among the female population in the post-2000 period using key determinants of health.

## Background

Overweight and obesity, defined by the World Health Organization as a body mass index (BMI) of 25.0–29.9 kg/m^2^ and ≥ 30.0 kg/m^2^ respectively, are the leading causes of coronary and ischaemic heart disease and other high‑risk non-communicable diseases [1–3]. Studying overweight and obesity together is important because both conditions significantly increase the risk of cardiovascular disease and other non-communicable diseases [4]. Among women of reproductive age, excess body weight is associated with increased risk of pregnancy complications such as pre-eclampsia, congenital anomalies, and stillbirth [5,6].

The prevalence of overweight and obesity continues to steadily rise on a global scale. In 2014, obesity among women and men surpassed underweight in 165 and 136 countries worldwide, with severe obesity exceeding in 135 countries among women and 113 countries among men [7]. While the burden remains highest in affluent regions such as the United States of America, Polynesia, Micronesia, the Middle East, Sub-Saharan Africa, and Central Europe, post-millennium trends indicate a rapid rise in overweight and obesity across low-and-middle-income (LMIC) regions, including Southeast Asia, Latin America, and the Caribbean [7–10].

Globally, women bear a disproportionately higher burden of obesity, with the number of women living with obesity increasing by more than five times over recent decades, with an estimated rise of 321 million obese adult women from 1975 to 2016 [7,8,11–13]. Sex-disaggregated data from several South Asian countries show that prevalence rates of obesity were more than double those of men in 2022 [14]. The higher prevalence of overweight and obesity among women may reflect a combination of biological and social influences. Physiological changes related to pregnancy, childbirth, and hormonal variation can increase susceptibility to weight gain, while gendered roles and caregiving responsibilities may reduce opportunities for physical activity [15]. In many settings, women also face structural constraints in access to employment and decision-making, which can shape dietary choices and long-term nutritional outcomes [16].

A landmark Lancet study reported a consistent increase in the mean BMI of women in Southeast Asian countries by over 1 kg/m^2^ per decade, with a more pronounced surge after 2000 compared with earlier periods. By 2014, an estimated 375 million women globally were reported obese [7]. Projections estimate that low-middle-income countries like Bhutan will bear the highest future burden of overweight and obesity in the region, with over 69 million women at risk of obesity complications by 2030 in the Southeast Asian region [17].

These shifts have contributed to the emergence of the double burden of malnutrition (DBM), characterized by the simultaneous presence of undernutrition and overweight or obesity within the same countries, communities, households, and individuals [11,13]. The coexistence underscores the complexity of nutritional challenges in South Asia, where longstanding structural drivers of malnutrition are layered upon poverty, gender inequality, and limited access to health services. In South Asia, this phenomenon also reflects rapid socioeconomic and dietary transitions occurring alongside food insecurity and inequitable access to nutritious diets. Historically dominated by undernutrition, South Asian health profiles have changed markedly over recent decades. Understanding trends in overweight and obesity is therefore critical not only for tracking the growing burden of NCDs but also for contextualising these patterns within the broader DBM. Trend analyses provide insight into how nutritional risks evolve over time and identify population subgroups disproportionally affected by both forms of malnutrition.

Studies consistently have shown that overweight and obesity vary by age, gender, place of residence, education level, and wealth quintile [11,13]. Other studies discuss determinants for the obesity epidemic to be due to the food environment, supply of energy-dense foods combined with sedentary lifestyles [8,11,13,18]. The DBM demonstrates nutritional and socio-economic transitions occurring within similar households and populations. Examining socio-economic inequalities in overweight and obesity as well as undernutrition is therefore vital for pinpointing structural inequities, understanding the complex factors driving divergent nutritional outcomes, and designing effective, targeted interventions [19].

This study aims to analyse and compare the trends of prevalence in overweight and obesity across five selected South Asian countries with repeated Demographic Health Survey (DHS) anthropometric data over the post-millennium period (2000–2022) focusing on three social determinants: household wealth quintile, education attainment, and urban-rural residence. Although this study focuses on overweight and obesity rather than modelling undernutrition, it contextualizes these trends within the broader DBM in South Asia and informs equity-focused health policies and resource allocation for and health promotional programs across South Asia.

## Methods

### Study design

The study analysed DHS data, carried out between 2000 and 2022 from five countries in the South Asian region selected based on the data availability for the variables of interest: Bangladesh, India, the Maldives, Nepal, and Pakistan. The DHS surveys are nationally representative cross-sectional household surveys that collect data for a wide range of population health indicators including nutritional status of men and women in low-to-middle-income countries.

The DHS surveys maintain uniform methodologies for gathering data and employ similar questionnaires, enabling easy comparison across different countries. The surveys employ a standardised multistage cluster sampling design to generate nationally representative estimates at the time of each survey. In brief, clusters (primary sampling units defined by census geographic areas) are first selected from nationally defined sampling frames, stratified by urban and rural residence. In the second stage, households are systematically sampled within each selected cluster, and all eligible women aged 15–49 years are invited to participate in individual interviews. Further details of the survey design, training procedures, data processing, and dissemination phases are described in DHS technical documentation [20].

### Study population

We selected all surveys post 2000 in the five selected countries including India (2005, 2015, 2021), Bangladesh (2000, 2004, 2007, 2011, 2014, 2017, 2022), Pakistan (2012, 2017), Maldives (2009, 2016) and Nepal (2001, 2006, 2011, 2016, 2022). Data collection focused on women of reproductive age (15–49 years old). Women who gave birth within 2 months prior to the survey were excluded. We extracted the women’s records (IR) of each country and appended them.

### Ethical considerations

All DHS surveys are conducted in accordance with strict ethical standards. Informed consent is obtained from all participants prior to data collection, with respondents clearly informed about the purpose of the survey, the right to refuse or withdraw at any time without consequence. All data are collected privately and de-identified before being made available for secondary analysis. Ethical approval for DHS survey protocols was obtained by the Inner City Fund Institutional Review Board and by relevant national ethics committees in each participating country.

### Data collection instruments

The outcome variable for the current study was overweight and obesity in women of reproductive age (15–49 years). Overweight or obesity were defined as BMI ≥ 25 kg/m^2^, thus including both overweight (BMI 25–29.9 kg/m^2^) and obesity (BMI ≥ 30 kg/m^2^). BMI levels were calculated and recorded as weight in kilograms divided by height in metres squared in accordance with the World Health Organisation (WHO) guidelines [21]. There are studies that have used reduced BMI thresholds (e.g. ≥23 kg/m^2^ for overweight), which have been proposed for South Asian populations due to their higher cardiometabolic risk at lower BMI levels [22]. However, these lower cut-offs were not applied in the present study as our analysis is explicitely comparitive across countries over time and the WHO standard cut-off BMI classification as a basis for global NCD and SDG monitoring, permitting our estimates to be read against international literature. Although lower thresholds may improve sensitivity for identifying risk within specific populations, the use of globally standardised cut-offs strengthens methodological consistency and the validity of cross-country comparisons. Specifically, the two specifications yield the same substantive conclusions. Both thresholds increase monotonically with the direction and approximate magnitude of change overtime is preserved.

The DHS Program applies standardized survey procedures across countries, including sampling methods, questionnaires, anthropometric measurement protocols, interviewer training, field supervision, and data-quality checks, supporting the validity, reliability, and comparability of the data. In the present analysis, consistent variable definitions, eligibility criteria, and analytical procedures were applied across all countries. No country-specific adjustment of the original data was undertaken, and standard DHS sampling weights were applied to account for the complex survey design and generate nationally representative estimates.

This study was theoretically informed by the nutrition transition, which refers to the long-term shift toward energy-dense diets and more sedentary lifestyles accompanying urbanisation and socioeconomic change, resulting in rising overweight and obesity. The analysis was further guided by the UNICEF 2020 Conceptual Framework for Maternal and Child Nutrition and the WHO Conceptual Framework for Action on the Social Determinants of Health [23,24], which together provide a logic linking structure and socioeconomic conditions to intermediate behavioural pathways (e.g. diet, physical activity, access to resources) which in turn influence nutritional outcomes. Guided by these conceptual frameworks, trends in overweight and obesity were examined across key social determinants of health, including household wealth, women’s education level and place of residence.

We also examined the trend of overweight and obesity across different social determinants of health including household wealth index, women’s level of education and the place of residence. The household wealth index, a gauge of socio-economic status, was generated through each survey’s principal component analysis, encompassing various indicators like asset ownership, housing type, sanitation services, and water access [20]. Based on the household wealth index, households were classified into quintiles, with the first quintile (Q1) being the poorest group and the fifth quintile (Q5) being the richest group. The level of education was categorized into two levels: No education and higher education. The lowest and the highest education groups were included in this analysis to show magnitude and absolute gaps in inequity. Lastly, place of residence was categorized into urban or rural setting.

### Data analysis

The survey command (svy) was used to report for the effect of sampling weights, accounting for valid statistical inference, weighted sample distribution and reducing bias from non-response or non-sampling errors. DHS surveys involved two stage household cluster sampling procedure in all analyses. The first stage of cluster sampling involved selecting households independently and proportionately to their geographic area. The second stage uses systematic sampling to select households with equal probability. The absolute difference (AD) and average annual rate of change (AARC) in the prevalence of overweight or obesity were estimated across countries, stratified by social determinants with corresponding confidence intervals, following approaches used in prior epidemiological analyses [25–27]. AD was calculated as the latest available overweight and obesity prevalence minus the earliest available prevalence for each country or subgroup. The AARC in overweight and obesity was estimated using regression-based methods. Where prevalence estimates were available for multiple survey years, log regression models were estimated separately within each social determinant (e.g. education attainment level). This was calculated using the following formula

$$\mathrm{AARC}=\left(\exp\left(\beta \right)-1\right)\times 100$$

β symbolizes the coefficient derived from a simple linear regression analysis, representing the average annual proportion change in prevalence. This coefficient is obtained by regressing the natural logarithm of prevalence ln(Yi) against time (ti). This approach assumes a constant rate of change in prevalence over time and allows for comparability of trends across countries and population subgroups. The analysis did not include multivariable adjustment limits, thus interpretation of causality or independent effects from the patterns observed cannot be determined. The statistical software used was Stata 16.1 (StataCorp, College Station, Texas, USA) under license by the University of Sydney.

## Results

Our final dataset comprised 1,561,090 women of reproductive age from 19 survey datasets (see Table 1 below): Bangladesh (7), India (3), Nepal (5), Maldives (2) and Pakistan (2). These surveys spanned a period of 22 years in Bangladesh and Nepal, 18 years in India, 10 years in the Maldives and 6 in Pakistan. The earliest data points were from Bangladesh and Nepal in 2000, while the most recent were collected in 2022 from Bangladesh, Nepal, and India. There were seven survey rounds conducted in Bangladesh, five in Nepal, three in India, and two in both the Maldives and Pakistan. The sample size in different round of surveys ranged from 3,722 in Pakistan in 2016–2017 to 660,741 in Indian in 2014–2015.

**Table 1.** Sample size of DHS datasets by country and survey period, total *n* = 1,561,090 women aged 15–49 years across 19 country-survey datasets.

	2000–2001	2004–2006	2007–09	2011–2012	2014–2015	2016–2017	2021–22
Bangladesh	4,510	10,431	10,021	16,024	16,478	18,328	9,213
India	–	111,781	–	–	647,168	–	660,741
Nepal	7,784	10,003	–	5,800	–	6,069	7,007
Maldives	–	–	5,173	–	–	6,667	–
Pakistan	–	–	–	4,170	–	3,722	–

The overall trend across survey rounds for overweight and obesity prevalence in the five study countries is presented in bivariate scatter plot (Figure 1) and Table 2. We found a marked increase in the prevalence of overweight and obesity across all the studied countries, except for the Maldives. In Bangladesh between 2000 and 2006, the prevalence of overweight and obesity most substantially increased from 4.5% to 8.8% and between 2016 and 2022, and it ranged from 32.1% to 36.5%, resulting in an AD of more than a quarter (32.0%) between 2000 and 2022 and an annual average increase of 10.1%. During the same period, Nepal reported an AD of 22.9% with an annual average increase of 8.0%. India and Pakistan reported very similar increases, with an AARC of 5.2% and 5.3%, respectively, and the Maldives reported the most modest increase with an AD of 4% between 2007 and 2016, and an AARC of 1.2%.

**Figure 1.**
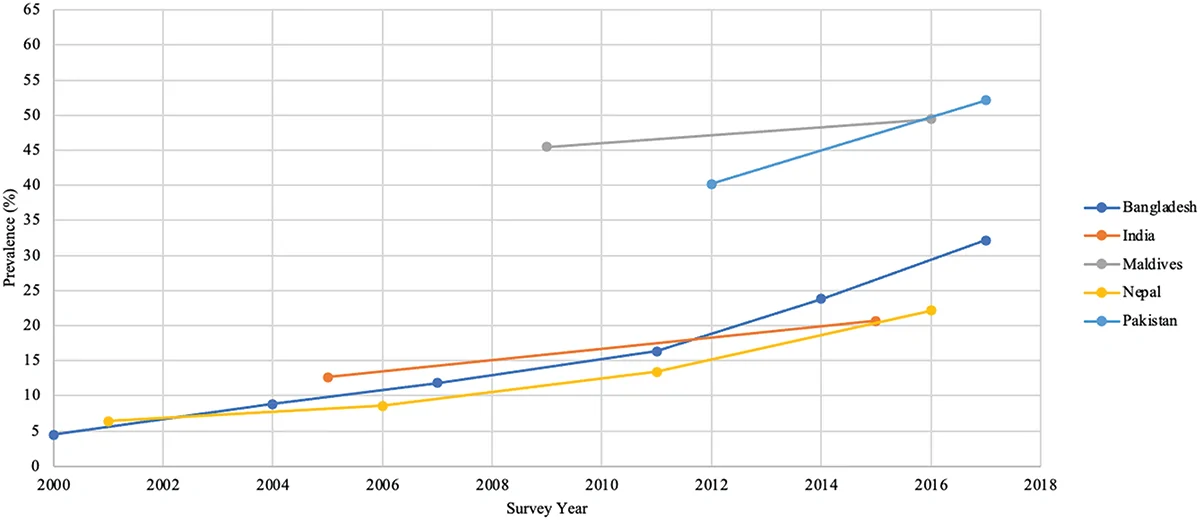
Lined scatter plot with dots showing overweight and obesity prevalence (BMI ≥ 25 kg/m^2^) among women aged 15–49 years in five selected South Asian countries, 2000–2022.

**Table 2.** The overall prevalence, AD, AARC and direction of trend of overweight and obesity among women of reproductive age in South Asian countries with confidence intervals, *n* = 1,561,090.

	2000–2001	2004–2006	2007–09	2011–2012	2014–2015	2016–2017	2021–22	AD	AARC	Direction
Bangladesh	4.48 (3.72–5.40)	8.82 (7.80–9.95)	11.83 (10.61–13.17)	16.36 (15.39–17.39)	23.77 (22.29–25.31)	32.13 (31.09–33.19)	36.48 (35.15–37.84)	32.00	10.08	↑
India	–	12.65 (12.17–13.14)	–	–	20.67 (NA)	–	24.02 (23.76–24.28)	11.37	5.15	↑
Maldives	–	–	45.47 (43.24–47.73)	–	–	49.44 (47.68–51.19)	–	3.97	1.20	→
Nepal	6.37 (5.34–7.59)	8.54 (7.25–10.03)	–	13.38 (11.77–15.17)	–	22.11 (20.36–23.97)	29.26 (27.28–31.34)	22.89	7.97	↑
Pakistan	–	–	–	40.19 (37.21–43.23)	–	52.13 (48.83–55.40)	–	11.94	5.34	↑

Note: *AD: Absolute Difference, this was calculated as the latest available prevalence minus the earliest available prevalence for each country. AARC: Average Annual Rate of Change, this was estimated using regression-based methods (see Methodology section for more detail), Direction: upward arrow = increasing AARC; downward arrow = decreasing AARC; flat arrow = no trend.

Figure 2 and Table 3 present the prevalence of overweight and obesity across urban and rural settings in the five countries studied. In countries with data from 2000 to 2006, the prevalence in rural settings ranged from 2.6% in Bangladesh to 7.4% in India, while in urban settings it ranged from 13.1% in Bangladesh to 24.1% in Nepal. Subsequently, in countries with data from 2021 to 22, the prevalence in rural settings ranged from 19.7% in India to 32.9% in Bangladesh, whereas in urban settings it ranged from 32.7% in Nepal to 45.5% in Bangladesh. The AARC across the five studied countries varied from 2.9% in the Maldives to 12.4% in Bangladesh in rural settings and from a negative 1.6% in the Maldives to 5.9% in Bangladesh in urban settings.

**Figure 2.**
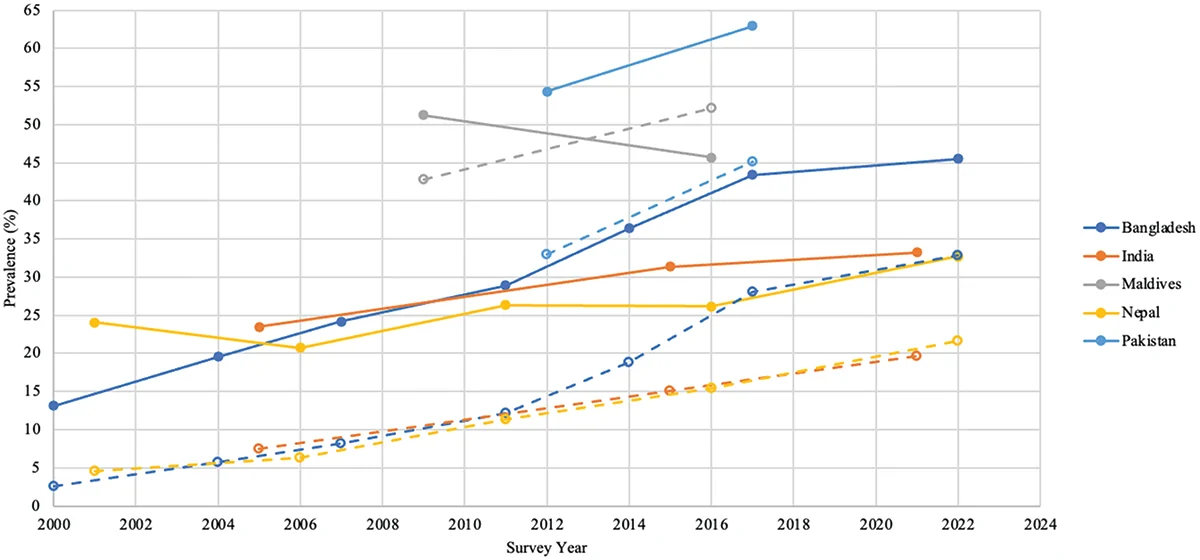
Lined scatter plot with dots showing overweight and obesity prevalence (BMI ≥ 25 kg/m^2^) across place of residence (solid lines denote urban areas, dashed lines denote rural areas) among women aged 15–49 years in five selected South Asian countries, 2000–2022.

**Table 3.** The prevalence, AD, AARC and direction of trend of overweight and obesity across place of residence among women of reproductive age in South Asian countries with confidence intervals, *n* = 1,561,090.

Place of residence	2000–2001	2004–2006	2007–09	2011–2012	2014–2015	2016–2017	2021–22	AD	AARC	Direction
Bangladesh										
Urban	13.13 (10.60–16.17)	19.54 (16.72–22.71)	24.19 (21.34–27.29)	28.90 (26.47–31.36)	36.39 (33.79–39.07)	43.41 (41.47–45.38)	45.53 (42.73–48.36)	32.40	5.91	↑
Rural	2.59 (2.03–3.30)	5.75 (4.98–6.63)	8.16 (7.13–9.33)	12.11 (11.11–13.19)	18.79 (17.27–20.42)	28.03 (26.81–29.30)	32.85 (31.4–34.33)	30.26	12.37	↑
India										
Urban	–	23.48 (22.57–24.43)	–	–	31.35 (30.82–31.88)	–	33.26 (32.72–33.81)	9.78	2.80	↑
Rural	–	7.43 (7.09–7.78)	–	–	15.06 (14.83–15.29)	–	19.68 (19.43–19.93)	12.25	7.84	↑
Maldives										
Urban	–	–	51.25 (46.29–56.19)	–	–	45.67 (42.33–49.05)	–	−5.58	−1.63	↓
Rural	–	–	42.75 (40.79–44.74)	–	–	52.11 (50.40–53.82)	–	9.36	2.87	↑
Nepal										
Urban	24.07 (19.97–28.70)	20.74 (17.83–23.99)	–	26.34 (23.05–29.91)	–	26.16 (23.70–28.78)	32.73 (30.11–35.46)	8.66	1.67	↑
Rural	4.53 (3.67–5.57)	6.27 (5.04–7.79)	–	11.38 (9.65–13.37)	–	15.44 (13.26–17.90)	21.67 (19.66–23.83)	17.14	8.03	↑
Pakistan										
Urban	–	–	–	54.34 (51.04–57.61)	–	62.94 (59.13–66.59)	–	8.60	2.98	↑
Rural	–	–	–	33.01 (29.81–36.37)	–	45.17 (40.73–49.70)	–	12.16	6.47	↑

Note: *AD: Absolute Difference, was calculated as the latest available prevalence minus the earliest available prevalence for each country. AARC: Average Annual Rate of Change, this was estimated using regression-based methods (see Methodology section for more detail), Direction: upward arrow = increasing AARC; downward arrow = decreasing AARC; flat arrow = no trend.

Figure 3 and Table 4 present the prevalence of overweight and obesity among women with secondary or higher education compared with those with low or no education. Between 2000 and 2006, rates ranged from 4.2% in Nepal to 26.8% in Bangladesh among women with secondary or higher education, while those with low or no education ranged from 2.1% in Bangladesh to 19.3% in Nepal. Over the following decade, a notable increase in the prevalence of overweight and obesity was observed in both educational groups. In 2021–22, prevalence of women with secondary or higher education ranged from 25.23% in India to 41.10% in Bangladesh, while those with low or no education, it ranged from 21.55% in India to 28.15% in Bangladesh and a remarkable 69.97% in the Maldives for years 2016–17.

**Figure 3.**
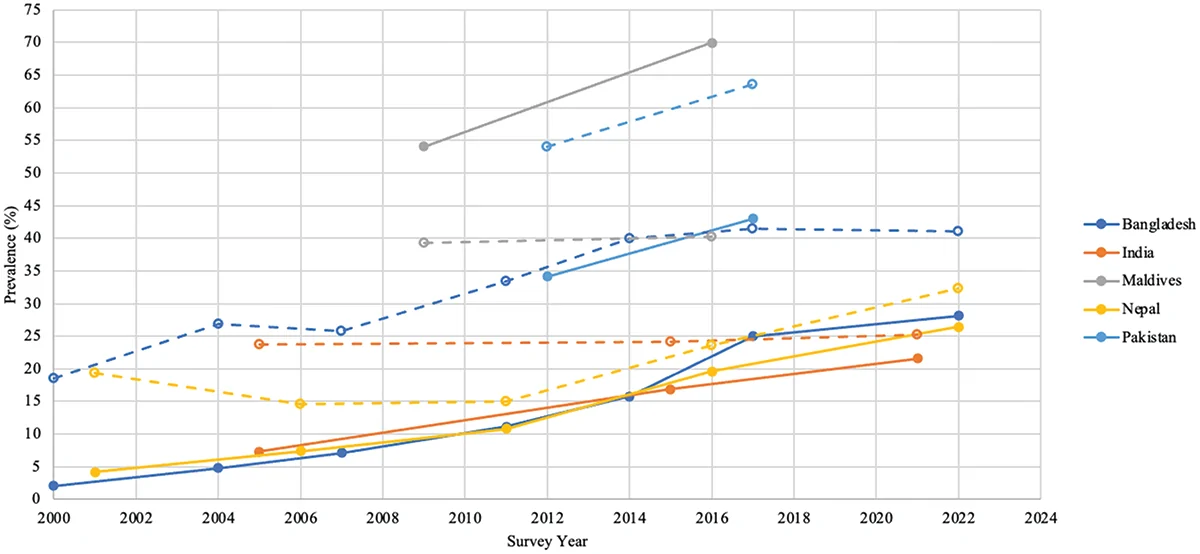
Lined scatter plot with dots showing overweight and obesity prevalence (BMI ≥ 25 kg/m^2^) across education level (solid lines denote no education areas and dashed lines denote higher education) among women aged 15–49 years in five selected South Asian countries, 2000–2022.

**Table 4.** The prevalence, AD, AARC and direction of trend of overweight and obesity across level of education among women of reproductive age in South Asian countries with confidence intervals, *n* = 1,561,090.

Education level	2000–2001	2004–2006	2007–09	2011–2012	2014–2015	2016–2017	2021–22	AD	AARC	Direction
Bangladesh										
None	2.08 (1.50–2.86)	4.77 (4.04–5.61)	7.08 (6.11–8.18)	11.15 (10.04–12.35)	15.79 (13.73–18.10)	25.00 (23.22–26.87)	28.15 (25.43–31.04)	26.07	12.71	↑
High	18.53 (14.76–23.00)	26.82 (22.44–31.70)	25.80 (22.68–29.18)	33.35 (30.35–36.49)	39.99 (36.98–43.08)	41.45 (39.32–43.61)	41.10 (38.6–43.4)	22.57	3.78	↑
India										
None	–	7.28 (6.88–7.71)	–	–	16.84 (16.51–17.17)	–	21.55 (21.19–21.91)	14.27	8.88	↑
High	–	23.78 (22.57–25.02)	–	–	24.20 (23.67–24.73)	–	25.23 (24.75–25.71)	1.45	0.41	→
Maldives										
None	–	–	54.04 (50.78–57.27)	–	–	69.97 (62.42–76.57)	–	15.93	3.76	↑
High	–	–	39.33 (32.04–47.13)	–	–	40.24 (36.55–44.04)	–	0.91	0.33	→
Nepal										
None	4.19 (3.41–5.14)	7.37 (6.13–8.82)	10.78 (8.77–13.18)	–	–	19.57 (17.34–22.02)	26.39 (23.63–29.34)	22.20	9.33	↑
High	19.31 (14.39–25.4)	14.54 (12.11–17.36)	15.05 (13.10–17.24)	–	–	23.60 (20.70–26.75)	32.37 (28.71–36.26)	13.10	3.02	↑
Pakistan										
None	–	–	–	34.15 (30.80–37.65)	–	43.02 (39.27–46.85)	–	8.87	4.73	↑
High	–	–	–	54.01 (49.73–58.22)	–	63.57 (58.69–68.18)	–	9.56	3.31	↑

Note: *AD: Absolute Difference, was calculated as the latest available prevalence minus the earliest available prevalence for each country. AARC: Average Annual Rate of Change, this was estimated using regression-based methods (see Methodology section for more detail), Direction: upward arrow = increasing AARC; downward arrow = decreasing AARC; flat arrow = no trend.

Figure 4 and Table 5 show the prevalence of overweight and obesity across the highest- and lowest-wealth quintiles. From 2004 to 2006, the prevalence in the poorest households ranged from 1.8% in India to 2.5% in Nepal, whereas in the wealthiest households, it varied from 23.0% in Nepal to 30.5% in India. In countries with 2021 data from the lowest wealth quintiles, the prevalence ranged from 10.0% in India to 21.2% in Bangladesh. Concurrently, those in the highest wealth quintile had prevalence rates ranging from 38.6% in India to 53.5% in Bangladesh, with a very high 72.7% in Pakistan from the years 2016–17. From this we can gather that while women in wealthier households still had a higher prevalence, AARC values indicate faster increases among women in poorer households, especially in Bangladesh, Nepal and India.

**Figure 4.**
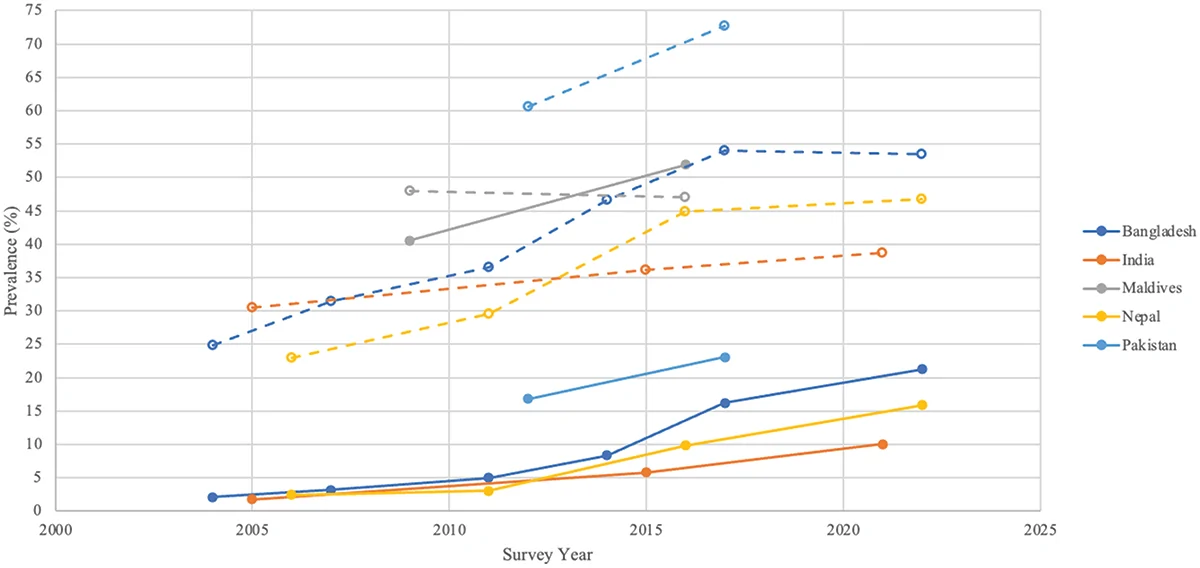
Lined scatter plot with dots showing overweight and obesity prevalence (BMI > 25 kg/m²) across wealth quintile (solid lines denote poorest quintile, dashed lines denote richest quintile) among women aged 15-49 years in five selected South Asian countries, 2000-2022.

**Table 5.** The prevalence, AD, AARC and direction of trend of overweight and obesity across wealth quintile among women of reproductive age in South Asian countries with confidence intervals, *n* = 1,561,090.

Wealth quintile	2000–2001	2004–2006	2007–09	2011–2012	2014–2015	2016–2017	2021–22	AD	AARC	Direction
Bangladesh										
Wealthiest	–	24.79 (22.17–27.60)	31.39 (28.79–34.15)	36.52 (34.26–38.85)	46.67 (44.27–49.08)	53.96 (51.91–56.00)	53.50 (50.47–56.51)	28.71	4.65	↑
Poorest	–	2.12 (1.51–2.95)	3.21 (2.39–4.30)	4.96 (4.07–6.05)	8.35 (7.00–9.94)	16.22 (14.86–17.69)	21.22 (19.06–23.56)	19.10	14.55	↑
India										
Wealthiest	–	30.47 (29.56–31.40)	–	–	36.17 (35.59–36.75)	–	38.63 (38.03–39.23)	8.16	1.85	↑
Poorest	–	1.80 (NA)	–	–	5.83 (5.64–6.03)	–	10.00 (9.73–10.29)	8.20	14.07	↑
Maldives										
Wealthiest	–	–	48.01 (41.78–54.30)	–	–	46.99 (42.46–51.56)	–	−1.02	−0.33	→
Poorest	–	–	40.56 (37.12–44.09)	–	–	51.88 (49.01–54.74)	–	11.32	3.58	↑
Nepal										
Wealthiest	–	23.02 (20.17–26.14)	–	29.51 (26.20–33.04)	–	44.86 (40.81–48.98)	46.74 (42.84–50.68)	23.72	3.71	↑
Poorest	–	2.45 (1.63–3.67)	–	3.05 (2.08–4.45)	–	9.83 (7.89–12.17)	15.88 (13.79–18.21)	13.43	13.60	↑
Pakistan										
Wealthiest	–	–	–	60.58 (56.43–64.59)	–	72.67 (67.85–77.02)	–	12.09	3.71	↑
Poorest	–	–	–	16.79 (12.82–21.69)	–	23.04 (17.91–29.11)	–	6.25	6.53	↑

Note: *AD: Absolute Difference, was calculated as the latest available prevalence minus the earliest available prevalence for each country. AARC: Average Annual Rate of Change, this was estimated using regression-based methods (see Methodology section for more detail), Direction: upward arrow = increasing AARC; downward arrow = decreasing AARC; flat arrow = no trend.

## Discussion

### Overall trends: steadily increasing

The study revealed a noticiable increase in the prevalence of overweight and obesity in South Asian low-to middle-income countries. These findings are consistent with previous literature. The NCD Risk Factor Collaboration reported accelerated increases in BMI across South Asia for both sexes from 1975 to 2016 [28]. Meanwhile, Stevens et al. discovered that obesity prevalence nearly doubled between 1980 and 2008 and a global shift from underweight to obesity occurred since 1990 [10,18]. Together these studies provide continued evidence that overweight and obesity constitute a global public health concern [10,18,28]. More specifically, among women of reproductive age, overweight and obesity have increased significantly since the 2000s across similar socio-economic determinants, including urban–rural areas, education, and wealth [29–31]. A recent paper examining data from Central and Southeast Asia also found a similar rising trend in female obesity. However, the paper did not examine obesity trends across different social determinants of health [32]. These findings contrast with those of pre-millennium studies that showed less significant increases in South Asian overweight and obesity rates [9,18].

### Urban-rural differences

Across the LMICs studied, the prevalence of overweight and obesity remained consistently higher in urban areas than in rural areas; however, the rate of increase in rural areas was notably faster in rural populations. These outcomes align with results from previous studies conducted in South Asian countries including Bangladesh, India, the Maldives, Pakistan, and Nepal [31,33–38]. Several factors may contribute to this emerging trend.

Rural populations often have limited access to diverse and healthy foods, particularly in remote mountainous or hilly areas, a contrast that has also been associated with undernutrition and low birth weights among mothers [39,40]. A plausible explanation is that urbanization, infrastructure change, food-system transformation, and occupational shifts may be altering diet and physical activity patterns; however, these pathways were not directly measured in this study. This is evident in the rate of female employment in rural agriculture decreasing by 10% from 2011 to 2025 [41]. Physically demanding jobs such as manual labour are increasingly being replaced by more sedentary forms of employment. This type of employment reduces overall energy expenditure and increases vulnerability to weight gain. Additionally, the increased availability of processed foods in rural areas has driven remarkable sales growth in South Asian countries [42]. Household consumption of processed foods is already high in Bangladesh, Indonesia, Nepal, and Vietnam, with projected high growth rates averaging to 63% in rural areas and 74% in urban areas [43]. This dietary shift and declines in physical activity are accelerating the rise in obesity in rural communities of LMICs [44]. These patterns highlight the need for place-based prevention strategies, indicating that regional policies and interventions targeting rural food environments, physical activity opportunities, and the penetration of ultra-processed foods may be critical to slowing the rapidly rising burden of overweight and obesity in rural communities within the study region.

### Socioeconomic disparities

Across countries studied, the prevalence of overweight and obesity remained higher among women in the wealthiest households, but the rate of increase was often faster among women in the poorest households. This suggests that the burden of disease is diffusing from higher socioeconomic groups towards poorer groups while the absolute prevalence gap remains large. These findings are consistent with previous studies conducted in Asian LMICs, including Bangladesh and India [45–48]. This pattern likely reflects the nutritional transitions accompanying economic development and urbanisation, which is associated with substantial shifts in dietary behaviours and lifestyle patterns. For example, between 1972 and 2001 in India, consumption of cereal grains declined among low-income groups while pulse consumption decreased across all income groups [49].

Economic development has also driven lifestyle changes, including reduced physical activity due to increasingly sedentary jobs and the convenience of urban transportation. When combined with higher-calorie diets, these behavioural shifts contribute to rising obesity prevalence. Women of low socioeconomic status generally have lower level of education, which, as reported here, are associated with an increased prevalence of overweight and obesity, potentially due to limited health literacy, reduced access to nutrition information, and constrained opportunities for healthy lifestyle choices. Conversely, women with higher education may obtain professional yet sedentary jobs and have greater exposure to westernized diets [48], both of which have strong potential to contribute to a higher BMI.

Together, these dynamics reflect the influence of wealthier urban environments that often encourage the consumption of fast foods and convenience foods that are rich in fats and sugars but low in nutrients, further contributing to overweight and obesity. This pattern has been well documented in India and developing countries, where rapid urbanization and rising incomes have led to accelerated dietary transitions [44,48].

Overall, these findings point to the importance of socio-economically differentiated interventions, suggesting that nutrition policies should address high current prevalence among wealthier and urban women while also preventing rapid increases among poorer and rural women as food environments and livelihoods change.

### Education and nutritional status

Educational attainment also played a significant role, with higher prevalence of overweight and obesity observed primarily among women with secondary or higher levels of education. These findings are consistent with national-level study conducted in India and Bangladesh [47]. In contrast, a multi-country analysis of 39 low-income countries reported an increased prevalence of overweight in groups with the lowest education level [47], highlighting hetergeneity in the eduction and obesity relationship across contexts. Education attainment is closely linked to household socioeconomic status and influences dietary choices, health literacy, and lifestyle behaviours. National policy interventions that strengthen consumer education such as including nutrition science in school curricula and providing nutrition counselling through health practitioners may reduce the prevalence rates of overweight and obesity among women with higher and lower levels of education attainment [42].

In addition, nutrition education programmes should increasingly focus on dietary quality, food literacy, and healthy lifestyle behaviours among populations undergoing rapid socioeconomic change. Strategies to promote regular physical activity and reduce sedentary behaviour may be particularly important among urban and socioeconomically advantaged groups, where increases in overweight and obesity were more pronounced.

### Country specific policy implications

Consequently, policy responses should reflect the country-specific patterns observed in this study and build on existing national policy platforms. In Bangladesh, the increase in overweight and obesity among urban and socioeconomically advantaged women supports strengthening multisectoral NCD actions that promote healthier food environments and more active urban settings, including improved opportunities for walking and cycling and reduced availability of foods high in fat, sugar, and salt [50].

In India, these findings support further integration of obesity prevention into the National Programme for Prevention and Control of Non-Communicable Diseases, including BMI screening, dietary counseling and promotion of physical activity through primary healthcare, alongside food-environment initiatives such as Eat Right India [51].

In Nepal, the Multi-Sector Nutrition Plan III (2023–2030) provides an appropriate platform for addressing the emerging double burden of malnutrition, complemented by stronger nutrition labelling, restrictions on unhealthy food marketing, and promotion of physical activity [52].

In Pakistan, where national policy already recognises the coexistence of maternal undernutrition, overweight, and obesity, greater emphasis should be placed on integrating healthy-diet and physical-activity interventions into services for women of reproductive age, particularly in urban populations, together with measures to improve the food environment [53].

The National Multi-Sectoral Action Plan for the Prevention and Control (2023–2031) in the Maldives plans to increase diet and physical activity and integrate a broad supply of environmental and infrastructure measures such as food access, urban walkability, and gym affordability. The National Plan also focuses on surveillance strategies that provide for broad population-wide measures and general equity principles yet shows a gap in disaggregated data infrastructure in specific social determinants [54]. These country-specific actions should complement continued efforts to address undernutrition among more disadvantaged population groups.

### Strengths and limitations

Several limitations should be considered when interpreting these findings. First, because the DHS surveys are repeated cross-sectional surveys, the associations reported here therefore describe co-occurrence rather than sequence, and no causal direction can be established between social determinants and nutritional status. Second, the residual confounding is likely. Although the models adjust for place of residence, wealth quartiles, and level of education, unmeasured factors including food security, health service quality, and intra-household food allocation may account for part of the observed associations. Thirdly, dietary intake and physical activity were not measured consistently across the included DHS surveys, thus their omission means the pathways influencing social determinants to the outcome could not be examined directly, and the estimated effects of the social variables may partly reflect these unmeasured factors. Fourthly, we couldn’t obtain comparable repeated DHS data for Afghanistan, Bhutan, and Sri Lanka due to the unavailability of anthropometric measurements. Afghanistan’s height and weight weren’t collected in surveys conducted in 2010 and 2015, while Bhutan was never a DHS program country and Sri Lanka’s anthropometric data hasn’t been collected since 1987. Fifthly, the availability of variables varied across countries, with DHS surveys typically conducted every 5 years but not systematically released in the same year in all countries. This inconsistency creates data gaps and asynchronous trend patterns, complicating comparative analyses. For the Maldives and Pakistan, and for some subgroup estimates, AARC was estimated from only two time points; these estimates should be interpreted cautiously because they cannot capture nonlinear trends. Finally, the use of cross-sectional data limits causal inference about the relationship between social determinants and nutritional status, as the analysis relies on population-level rather than individual-level associations, thereby increasing the risk of ecological fallacy.

## Conclusions

This study provides robust, nationally representative evidence that overweight and obesity among women of reproductive age have increased markedly across South Asian low-to-middle-income countries in the 21^st^ century, with the steepest increases observed among wealthier, more educated women and in rural populations experiencing rapid socioeconomic change. While urban areas continue to have the highest obesity rates, the accelerating rise in rural communities highlights the urgent need for place-based interventions.

Our findings underscore the need for equity-focused policies that address the socio-economic drivers of rising overweight and obesity, particularly those related to urbanisation, educational attainment, and household wealth. Based on these results, priority should be given to preventing further increases in overweight and obesity through tailored policy interventions targeting food systems and environmental factors.

## Supplementary Material

STROBE_nut_checklist_GHA.pdf

## Data Availability

The data used in this study are publicly available from the Demographic Health Survey Program funded by the United States Agency for International Development (USAID). DHS datasets are accessible upon request from the website https://dhsprogram.com/. Researchers must agree to the data use agreement and obtain approval prior to downloading the datasets. The DHS women’s nutrition indicator code used to derive BMI-related indicators is publicly available through the DHS Program GitHub repository. The study-specific analytic code used to append datasets, calculate prevalence, AD, and AARC, and generate tables/figures will be shared on reasonable request.
